# Copper Content Inversion of Copper Ore Based on Reflectance Spectra and the VTELM Algorithm

**DOI:** 10.3390/s20236780

**Published:** 2020-11-27

**Authors:** Yanhua Fu, Hongfei Xie, Yachun Mao, Tao Ren, Dong Xiao

**Affiliations:** 1 JangHo Architecture College, Northeastern University, Shenyang 110169, China; fuyanhua@mail.neu.edu.cn; 2 Information Science and Engineering School, Northeastern University, Shenyang 110004, China; 1870722@stu.neu.edu.cn; 3 School of Resources and Civil Engineering, Northeastern University, Shenyang 110819, China; maoyachun@mail.neu.edu.cn; 4 Software College, Northeastern University, Shenyang 110169, China; chinarentao@163.com

**Keywords:** copper ore, spectroscopy, extreme learning machine, VTELM, regularization

## Abstract

Copper is an important national resource, which is widely used in various sectors of the national economy. The traditional detection of copper content in copper ore has the disadvantages of being time-consuming and high cost. Due to the many drawbacks of traditional detection methods, this paper proposes a new method for detecting copper content in copper ore, that is, through the spectral information of copper ore content detection method. First of all, we use chemical methods to analyze the copper content in a batch of copper ores, and accurately obtain the copper content in those ores. Then we do spectrometric tests on this batch of copper ore, and get accurate spectral data of copper ore. Based on the data obtained, we propose a new two hidden layer extreme learning machine algorithm with variable hidden layer nodes and use the regularization standard to constrain the extreme learning machine. Finally, the prediction model of copper content in copper ore is established by using the algorithm. Experiments show that this method of detecting copper ore content using spectral information is completely feasible, and the algorithm proposed in this paper can detect the copper content in copper ores faster and more accurately.

## 1. Introduction

**Figure 1:**
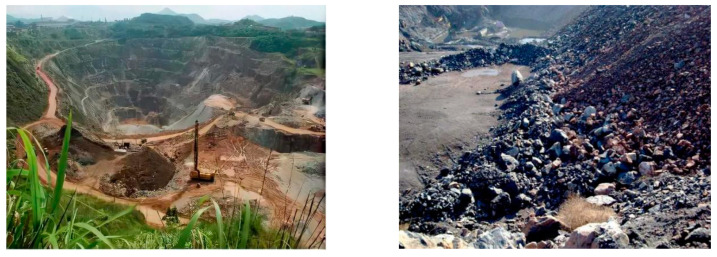
Open pit ore mining.

Copper is one of the oldest metals discovered by human beings. About 7000 years ago, casting of bronze ware appeared in Eurasia [1]. The proven reserves of copper resources in the world are 2.478 billion tons. The top ten countries with global copper reserves are Chile, the United States, Peru, Congo (Kinshasa), Australia, China, Russia, Mexico, Canada, and Argentina [2]. With the progress of the times, the continuous innovation of science and technology and the continuous development of world industrialization, the applications of copper are more and more extensive. As shown in Figure 1, open-pit ore mining situation is commonly used.

Recovery is an important index in mineral processing technology, which reflects the level of mineral processing technology and the quality of mineral processing work. For the copper content level in copper ore, the mineral processing technology adopted is not the same. Therefore, how to quickly and accurately measure the copper content in copper ore is particularly important. There are two kinds of traditional copper ore content determination [3,4,5], the first is the flame atomic absorption method, which has high accuracy, but low sensitivity and it is difficult to determine non-metallic elements by this technique. The second is to use chemical reagents to detect copper ores. Although this method has the same high accuracy, it is highly affected by the chemical reagents used, and the waste liquid causes great pollution. Therefore, how to measure copper content in copper ore stably and efficiently is a problem that must be solved in mineral processing technology, which is of great significance to reduce costs and improve efficiency. Spectral analysis technology is very mature, with the advantages of nondestructive testing, fast detection speed, high resolution, low cost. In recent years, it has become a trend to use spectral analysis instead of traditional analysis methods.

Mining in mining areas often leads to the destruction of the ecological environment, and it is extremely easy to change the nature of the soil on the surface and damage the health of surrounding residents. Therefore, the abandoned land after mining in the mining area should be reclaimed. Phytoremediation [6] can effectively absorb heavy metals or other toxic substances in the soil, thereby improving the soil. Luo [7] and others used commercial chrysanthemums to carry out heavy metal soil remediation. The results showed that after three years of soil remediation, the Cd in the soil decreased by 78.1%, and the Zn content decreased by 28.4%. In addition, the rice and green vegetables grown from the repaired soil meet the requirements of dietary safety. Saleem et al. [8] planted four kinds of jute products (HT, C-3, GC and SH) in the soil heavily polluted by copper to conduct experiments. The experiments show that C-3 and HT have the strongest survivability in copper-contaminated soil and may absorb Cu in the soil. Muro-Gonzalez et al. [9] conducted experiments under greenhouse conditions and found that *Prosopis laevigata* can grow normally under heavy metal pollution and is a potential plant for land reclamation. Chauhan and Mathur [10] proposed through experiments that sunflower can effectively remove heavy metals in contaminated soil.

Spectral analysis is a quantitative analysis method based on the principle of spectroscopy by detecting the characteristic wavelength and intensity of substances. Because it does not damage the sample during analysis, the operation is relatively simple, and the analysis speed is relatively fast, it has been widely used in many fields [11,12,13,14,15,16]. Because copper ore contains gangue minerals and other impurities, these impurities more or less affect the final results of spectral data. Therefore, the spectral data of copper ore contains many other spectral information related to the detection of copper ore content, which causes the spectral data of copper ore to have too high dimensions and redundant information. If the spectral data are directly used for modeling, the input dimension of the model will be too high, the amount of calculation will be large, and the structure will be too large. Therefore, the original spectral data of copper ore should be preprocessed, and dimensionality reduction is one of the most effective preprocessing methods for high-dimensional data. Dimension reduction can reduce the influence of noise on sample training as much as possible by removing the noise and redundant information in the data set, so as to simplify the training and prediction of machine learning models. As the data is compressed from high-dimensional to low-dimensional, the structure of the machine learning model will become simpler, the accuracy of training will increase and the time required for model training will also be greatly reduced.

Because the spectrum method can detect the sample quickly and without damage. So spectroscopy is more and more used in geology. Spectral technology can effectively identify minerals and quickly analyze minerals to identify exploration minerals [17]. Zhou et al. [18] used the spectral information of near standard soil samples to conduct hyperspectral modeling of lead content. The results show that this method can solve the problems of complex composition in soil and weak spectral information of heavy metals, and can further use remote sensing information to monitor soil heavy metals. Zhao et al. [19] established linear regression model and partial least square regression model by using the multispectral data obtained by UAV. The results show that the multispectral data can monitor the reclamation effect efficiently and quickly. Shin [20] and others studied the spectral information of heavy metal contaminated soil, and obtained that the spectral response of soil was positively correlated with the concentration of heavy metal. Zhang et al. [21] carried out four processing methods for the spectral data of heavy metal soil, and removed the noise of spectral information. Partial least squares regression and RBF neural network are used to model. The results show that the content of heavy metals in waste land can be effectively detected by using spectral information.

Principal component analysis (PCA) [22] is a simple and effective method for data processing, compression and dimensionality reduction [23,24,25]. It can use a small amount of data to retain the most important characteristics of the original data. PCA uses the direction of the largest variance of the original data as the projection, because the maximum variance of the data gives the most important information contained in the data. PCA can remove useless noise and reduce a lot of computation. Therefore, we used PCA to preprocess the original spectral data of copper ore.

Because machine learning models can process a large number of data, analyze and fit the data, and have strong generalization ability, in recent years, more and more researchers have begun to use and study machine learning, which leads to the continuous development of machine learning algorithms. Machine learning has made great progress in material science. Now more and more new materials cannot be found and designed without machine learning. Reference [26] proposes that in materials science, most of the existing experiments and computational modeling consume a lot of time and resources. Therefore, the use of machine learning to discover the properties of new materials, as well as the design and application of new materials, has been paid more and more attention. In this paper, the application of machine learning in material science is discussed in detail. Through the combination of material experiment and machine learning, a new idea is provided for the discovery of new material parameter performance. Reference [27] proposed that the discovery of new materials urgently needs to explore more advanced machine learning algorithms. Reference [28] summarized the application of machine learning in material design and material discovery of rechargeable batteries, discusses the problems of machine learning in the prediction of rechargeable battery performance, the structure and accuracy of machine learning models, and the problem of sample dimensions. Reference [29] discusses the importance of machine learning in the development and analysis of lithium sulfur batteries, and analyzes the key factors affecting lithium sulfur batteries by using machine learning method.

Extreme learning machine [30] (ELM) is a machine learning algorithm designed for single hidden layer feedforward neural network proposed by Huang in 2006. Since the algorithm randomly generates a hidden layer threshold and input weight, the algorithm does not need gradient back-propagation to adjust the weight. In this algorithm, the number of hidden layer neurons can be designed according to artificial experience or iterative optimization, and then the unique optimal output matrix can be solved by solving equations. Because of its simple structure and fast learning speed, more and more researchers begin to study this ELM algorithm deeply and use it in various fields [31,32,33,34]. Liang et al. proposed an online learning extreme learning machine (OS-ELM) by introducing an online learning mechanism into ELM [35]. This OS-ELM can update its own weights and thresholds with the training of new samples, without the need to retrain the model to obtain new weights and thresholds. Then, Huang et al. applied the integrated learning method to OS-ELM and proposed integrated online ELM (EOS-ELM) [36]. In 2016, Qu and Lang changed the single hidden layer of ELM to two hidden layers and proposed a two hidden layer extreme learning machine (TELM) [37]. In TELM, the number of neurons in the first hidden layer and the second hidden layer are the same, and the neuron connection method is the same as that of ELM, which is that the neurons are fully connected. In this paper, the two hidden layer Extreme learning machine with variable neuron nodes (VTELM) is proposed. The number of nodes in the middle two layers can be different, and the regularization standard is used to constrain the connection matrix between the second hidden layer and the output layer, which greatly improves the generalization ability of the Extreme learning machine.

The chapters of this paper are arranged as follows: the first chapter introduces the spectral analysis, principal component analysis and copper ore detection status. The second section introduces the preparation of copper ore samples and the process of spectral testing. In the third chapter, the algorithm flow of ELM and TELM is briefly described, and a two hidden layer extreme learning machine algorithm with variable hidden layer nodes (VTELM) is proposed. In the fourth part, the VTELM model is simulated and the simulation results are analyzed. Finally, the fifth part summarizes the main conclusions of this study.

## 2. Data Acquisition and Processing

All the copper ore samples are from the Deerni copper deposit (Qinghai Province, P.R. China) with a total of 251 samples. For copper ore samples, we used a SVC HR-1024 portable ground spectrometer (Vista Company, city, state abbrev, USA) for spectral testing. The parameters of the spectrometer are shown in Table 1.

**Figure 2:**
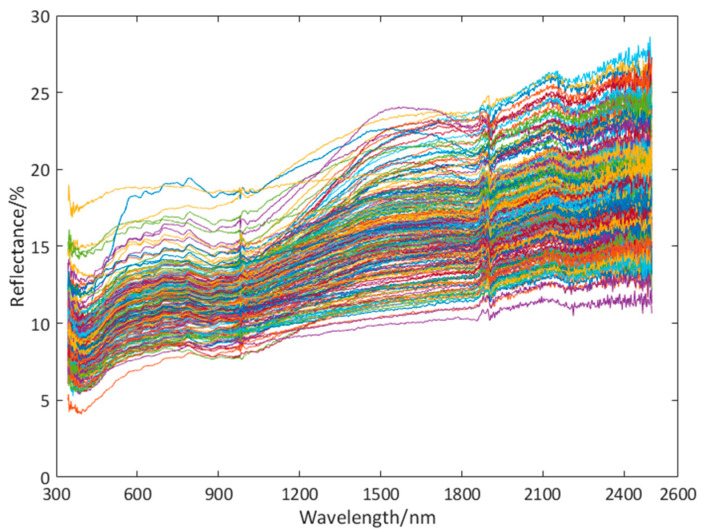
Spectrum of a copper ore.

Firstly, we clean and dry the copper ore samples collected, and then grind them. We chose to perform experiments from 10:00 to 14:00 during the day, while the weather was clear and cloudless. During the experiment, the probe of the spectrometer was perpendicular to the surface of the copper ore sample, and the distance between the probe and the sample surface should be 300 mm, the scanning time of the instrument was 1 s·times^−1^, and the whiteboard measurement value was corrected every 10 min. In order to reduce the measurement error, we conducted 10 spectral experiments on each sample of the spectrometer for three consecutive days. At the end of the experiment, the spectral information is collected, and the average value is taken as the spectral data (973 features). After the spectral test is completed, the spectral data of copper ore samples are obtained by preprocessing the measured spectral data such as band fitting. Figure 2 shows the spectrum of a copper ore.

## 3. Introduction to Neural Network

### 3.1. Extreme Learning Machine (ELM)

Single hidden layer Extreme learning machine is a kind of feedforward neural network. It consists of input layer, one hidden layer and output layer. For N arbitrary copper sample $\left(x_{i},t_{i}\right)$, where *x_i_* = [*x_i_*_1_*, x_i_*_2_,*…,x_in_*]*^T^*∈ *R^n^*, *t_i_* = [*t_i_*_1_*, t_i_*_2_,*…,t_im_*]*^T^*∈ *R^m^*, then the calculation formula of the extreme learning machine is as follows (Equation (1)):

(1) $$f_{L}(x)=\sum_{i=1}^{L}\beta_{i}g_{i}(x)=\sum_{i=1}^{L}\beta_{i}g(\omega_{i}*x_{j}+b_{i}),j=1,\ldots \ldots ,N$$

where *L* represents the number of neurons in the hidden layer of ELM; *N* represents the total number of samples entering the model training; *β_i_* represents the weight vector between the hidden layer and output layer; *ω_i_* is a random value, which represents the connection weight between the hidden layer neuron and the input feature; g represents the activation function; *b_i_* represents the offset vector; *x_j_* represents the input vector.

The calculation process of extreme learning machine is very similar to that of standard back propagation neural network, but the output matrix of hidden layer is pseudo inverse matrix. From Formula (1), we can draw the following conclusions:

(2) Hβ=T

In Equation (2):

(3) $$H={\left[\begin{array}{ccc} g(\omega_{1}*x_{1}+b_{1}) & ••• & g(\omega_{L}*x_{1}+b_{L})\\ \begin{array}{c} •\\ •\\ • \end{array} & ••• & \begin{array}{c} •\\ •\\ • \end{array}\\ g(\omega_{1}*x_{N}+b_{1}) & ••• & g(\omega_{L}*x_{N}+b_{L}) \end{array}\right]}_{N\times L}$$

(4) $$\beta ={\left[\begin{array}{l} \beta_{1}^{T}\\ \begin{array}{c} •\\ •\\ \begin{array}{l} •\\ \beta_{L}^{T} \end{array} \end{array} \end{array}\right]}_{L\times m}T={\left[\begin{array}{l} t_{1}^{T}\\ •\\ •\\ •\\ t_{N}^{T} \end{array}\right]}_{N\times m}$$

where: *m* is the number of outputs; *H* is the output matrix of hidden layer; *T* is the objective matrix of the training set.

Since the threshold of the hidden layer of ELM and the connection weight between the hidden layer and the input layer are randomly generated, the linear equation *Hβ* = *T* can be solved by the least squares method:

(5) Minimize:‖Hβ−T‖

Huang put forward two theorems on the basis of predecessors, and proved that the least square solution in the ELM model is:

(6) $$\overset{\wedge }{\beta }=H^{+}T$$

where *H*^+^ is the Moore Penrose generalized inverse of hidden layer output matrix **H**, and the least square solution of *Hβ* = *T* is unique.

### 3.2. Two Hidden Layer Extreme Learning Machine (TELM)

The TELM neural network is based on ELM, changing one hidden layer into two hidden layers. The number of neurons in the first layer and the first layer is the same, and the neurons in the previous layer are connected to each neuron in the next layer. During the operation of the TELM model, the connection weight between the first hidden layer and the input layer and the threshold value of the first hidden layer neuron are randomly selected, while the two hidden layer neurons The number can be obtained according to an empirical formula.

The TELM algorithm updates the output matrix of the second hidden layer by solving the connection weight of the first hidden layer and the second hidden layer and the threshold of the second hidden layer. Through calculation, the predicted output of TELM can be infinitely close to the actual output. TELM algorithm also has the advantages that ELM algorithm does not have, such as rarely falling into over fitting, updating the optimal solution and more suitable for big data processing. After algorithm analysis and actual analysis, TELM has unparalleled advantages in predictive regression and classification compared with ELM.

**Figure 3:**
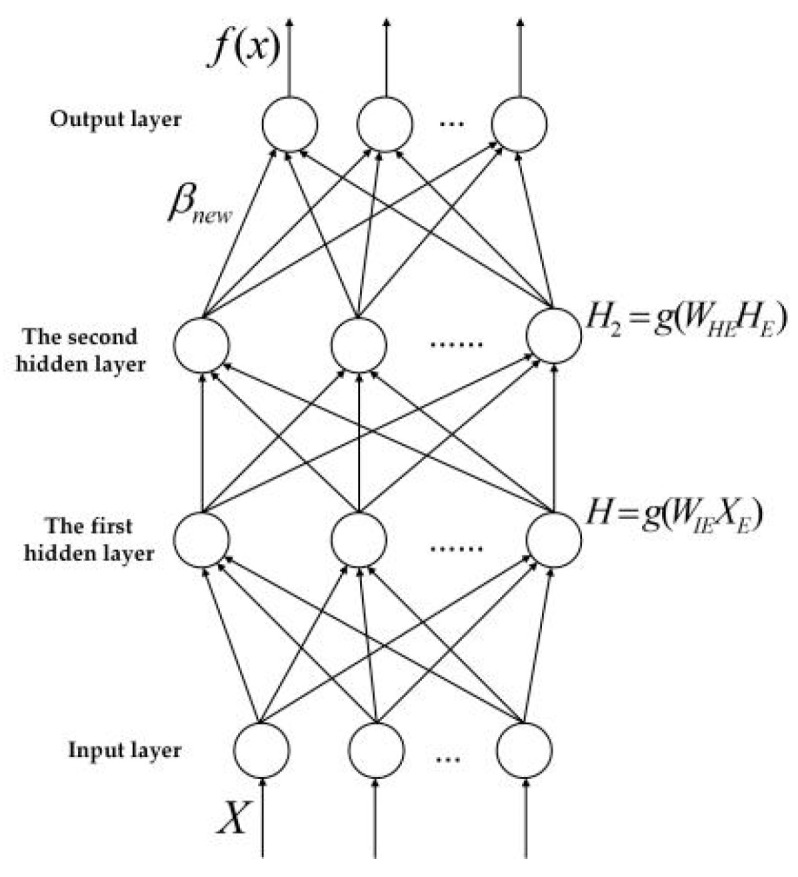
Network structure of TELM.

**Figure 4:**
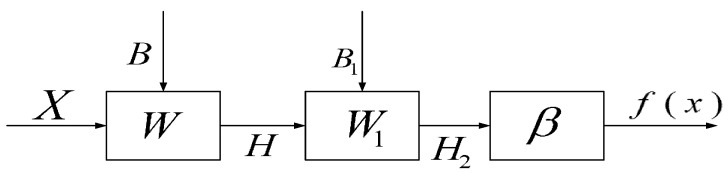
The work flow of the TELM.

TELM network structure diagram is shown in Figure 3, algorithm flow chart is shown in Figure 4. In the TELM algorithm, we mainly solve the value of parameters $W_{1},B_{1},\beta$.

Firstly, we assume that the training sample data set of TELM neural network is {*X,T*} *=* {*x_i_*,*t_i_*}(*i* = 1,2,…,*Q*), where *X* represents the input feature and *T* represents the feature. In TELM model, the first layer of hidden layer has the same activation function and the same number of hidden layer nodes as the second layer.

Then, in the algorithm design of TELM, the two hidden layers of TELM are regarded as one hidden layer, so TELM can be regarded as ELM. We can imitate ELM to get the output matrix *H* as follows:

(7) H=g(WX+B)

It can be seen from the workflow of the TELM that *W* and *B* are the weights and thresholds of hidden layers in ELM which are randomly initialized.

Next, by the ELM algorithm we can get matrix *β*:

(8) $$\beta =H^{+}T$$

Now, add a second hidden layer to the ELM algorithm, so that the ELM contains two hidden layers and restore it to TELM, and each hidden layer neuron is connected to each other, we can get the prediction output matrix of the second hidden layer *H*_1_ is:

(9) $$H_{1}=g(W_{1}H+B_{1})$$

Then the real output matrix *H*_1*_ of the second hidden layer is:

(10) $$H_{1*}=T\beta^{+}$$

where *β*^+^ is the generalized inverse of *β*.

Let *H*_1_ = *H*_1*_, which can maximize the predicted value of TELM close to the true value.

Now we assume the matrix $W_{HE}=[B_{1}W_{1}]$, so the weights *W*_1_ and threshold *B*_1_ of the second hidden layer can be solved as follows:

(11) $$W_{HE}=g^{-1}(H_{1*})H_{E}^{}{}^{+}$$

where $H_{E}^{+}$ is the generalized inverse of matrix *H_E_* = [1 *H*]^T^, 1 represents a Q-dimensional vector with each element 1. *g*^−1^(*x*) is the inverse function of *g*(*x*).

From Equation (11) we solve the parameters *W*_1_ and *B*_1_, then we can re-solve the second hidden layer prediction output *H*_2_:

(12) $$H_{2}=g(W_{1}H+B_{1})=g(W_{HE}H_{E})$$

Therefore, according to Figure 4 we can calculate *β* as:

(13) $$\beta_{\mathrm{new}}=H_{2}{}^{+}T$$

Finally, we can get the final output *f*(*x*) of the neural network as follows:

(14) $$f(x)=H_{2}\beta_{new}$$

In the calculation of *β*, when *β^T^β* is nonsingular, *β*^+^ = (*β^T^β*)^−1^*β^T^*.

### 3.3. Two Hidden Layer Extreme Learning Machine Algorithm with Variable Hidden Layer Nodes (VTELM)

The structure of the VTELM neural network is similar to the TELM structure. The difference is that the neuron nodes of each hidden layer of VTELM can be different from each other. In the process of VTELM model operation, because the connection weights of the first hidden layer and the input layer, the connection weights of the second hidden layer and the first hidden layer, as well as the threshold value of each hidden layer neuron are random values, we only need to set the nodes of the first layer hidden layer and the second layer hidden layer. The VTELM algorithm solves the connection weight between the output layer and the second hidden layer, which can make the final output of the VTELM neural network tend to the actual desired output result. And in this algorithm, only the neuron nodes of the first hidden layer and the second hidden layer need to be artificially set, and the optimal solution can be obtained without setting other parameters.

**Figure 5:**
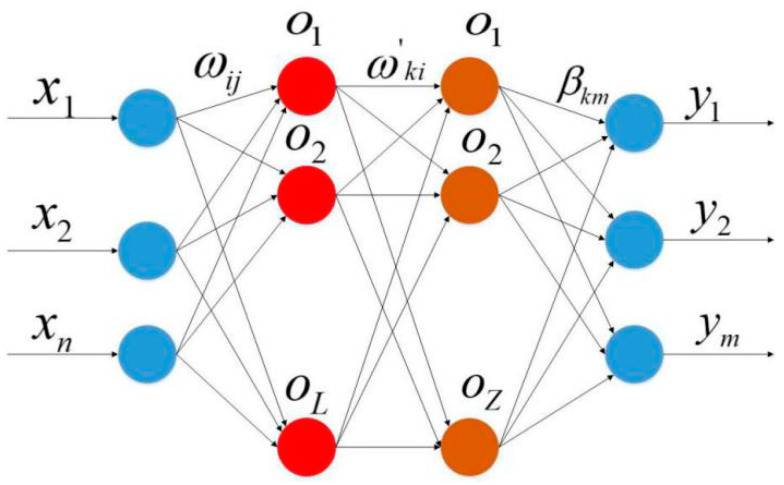
Network structure of VTELM.

**Figure 6:**
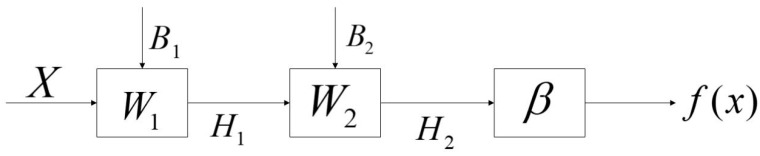
The work flow of the VTELM.

The network structure of the VTELM neural network is shown in Figure 5, and the algorithm flow can be represented in Figure 6. It can be seen from Figure 6 that VETLM only needs to solve the value of *β* to get the optimal output.

In Figure 5, {*x*_1_*, x*_2_,*…,x_n_*} represents the input characteristics of training samples, *ω_ij_* represents the connection weight between the *j*-th neuron node in the input layer and the *i*-th neuron node in the first hidden layer; *ω_ki_* represents the connection weight value between the *k*-th neuron node in the second hidden layer and the *i*-th neuron node in the first hidden layer; *β_km_* refers to the connection between the *k*-th neuron node in the second hidden layer and the *m*-th neuron node in the output layer weight; {*y*_1_*, y*_2_,*…,y_m_*} represents the output characteristics of training samples.

In VTELM algorithm, in order not to lose generality, suppose the connection weight *ω*_1_ between the first hidden layer and the input layer and the neuron threshold *b*_1_ of the first hidden layer are set as follows:

(15) $$w_{1}={\left[\begin{array}{cccc} \omega_{11} & \omega_{12} & ••• & \omega_{1n}\\ \omega_{21} & \omega_{22} & ••• & \omega_{2n}\\ \begin{array}{l} •\\ •\\ • \end{array} & \begin{array}{l} •\\ •\\ • \end{array} & ••• & \begin{array}{l} •\\ •\\ • \end{array}\\ \omega_{l1} & \omega_{l2} & ••• & \omega_{\ln} \end{array}\right]}_{l\times n}b_{1}={\left[\begin{array}{l} b_{1}\\ b_{2}\\ •\\ •\\ •\\ b_{l} \end{array}\right]}_{l\times 1}$$

Let the connection weight *ω*_2_ between the first hidden layer and the second hidden layer and the threshold *b*_2_ of neurons be set as:

(16) $$w_{2}={\left[\begin{array}{cccc} \omega_{11} & \omega_{12} & ••• & \omega_{1l}\\ \omega_{21} & \omega_{22} & ••• & \omega_{2l}\\ \begin{array}{l} •\\ •\\ • \end{array} & \begin{array}{l} •\\ •\\ • \end{array} & ••• & \begin{array}{l} •\\ •\\ • \end{array}\\ \omega_{z1} & \omega_{z2} & ••• & \omega_{zl} \end{array}\right]}_{z\times l}b_{2}={\left[\begin{array}{l} b_{1}\\ b_{2}\\ •\\ •\\ •\\ b_{z} \end{array}\right]}_{z\times 1}$$

The connection weight *β* between the second hidden layer and the output layer is:

(17) $$\beta ={\left[\begin{array}{cccc} \beta_{11} & \beta_{12} & ••• & \beta_{1m}\\ \beta_{21} & \beta_{22} & ••• & \beta_{2m}\\ \begin{array}{l} •\\ •\\ • \end{array} & \begin{array}{l} •\\ •\\ • \end{array} & ••• & \begin{array}{l} •\\ •\\ • \end{array}\\ \omega_{z1} & \beta_{z2} & ••• & \beta_{zm} \end{array}\right]}_{z\times m}$$

Firstly, the number of training samples is *Q*, the input eigenmatrix is X, and the expected output eigenmatrix is *Y*. There are *n* input features in the sample input, so there are *n* neuron nodes in the input layer of VTELM; There are *m* output features in the sample output, so there are *m* neuron nodes in the output layer of VTELM; Then assume that there are *l* neurons in the first hidden layer and *z* neurons in the second hidden layer, and the activation functions of the two layers are the same.

(18) $$X={\left[\begin{array}{cccc} x_{11} & x_{12} & ••• & x_{1Q}\\ x_{21} & x_{22} & ••• & x_{2Q}\\ \begin{array}{l} •\\ •\\ • \end{array} & \begin{array}{l} •\\ •\\ • \end{array} & ••• & \begin{array}{l} •\\ •\\ • \end{array}\\ x_{n1} & x_{n2} & ••• & x_{nQ} \end{array}\right]}_{n\times Q}\;\;\;\;\;\;Y={\left[\begin{array}{cccc} y_{11} & y_{12} & ••• & y_{1Q}\\ y_{21} & y_{22} & ••• & y_{2Q}\\ \begin{array}{l} •\\ •\\ • \end{array} & \begin{array}{l} •\\ •\\ • \end{array} & ••• & \begin{array}{l} •\\ •\\ • \end{array}\\ y_{m1} & y_{m2} & ••• & y_{mQ} \end{array}\right]}_{m\times Q}$$

Then, in the algorithm design of VTELM, the connection weight *ω*_1_ between the first hidden layer and the input layer and the neuron threshold *b*_1_ of the first layer are initialized randomly, then the output matrix *H*_1_ of the first hidden layer is:

(19) $$H_{1}=[g(\omega_{1}*x)+b_{1}]$$

The weights *ω*_2_ of the connection between the second hidden layer and the first layer and the threshold value *b*_2_ of the second layer neurons are initialized randomly. Then, *H*_1_ is used as the input matrix of the second hidden layer to calculate the output matrix *H*_2_ of the second hidden layer:

(20) $$H_{2}=[g(\omega_{2}*H_{1})+b_{2}]$$

Therefore, parameter *β* is:

(21) $$H_{2}^{}\beta_{}=T^{}$$

It can be concluded that:

(22) $$\overset{\wedge }{\beta }=H_{2}^{+}T$$

In order to prevent the output from overfitting, improve the robustness and generalization performance of the network, and make the network more stable, a regularization term is added to the solution process *β* to constrain [38,39]. It can be expressed as:

(23) $$\underset{\beta }{\arg\min E(W)}=\underset{\beta }{\arg\min}(\frac{1}{2}{\left\|\beta \right\|}^{2}+\frac{1}{2}\lambda {\left\|\varepsilon \right\|}^{2}),\;\mathrm{s.t.}\sum_{i=1}^{Z}\beta_{i}g(\omega_{i}•x_{j}+b_{i})-t_{j}=\varepsilon_{j},j=1,2,\ldots Q$$

In Equation (23), the sum of squares of ${\left\|\varepsilon \right\|}^{2}$ error represents empirical risk; ${\left\|\beta \right\|}^{2}$ represents structural risk, which originates from the principle of maximizing marginal distance in statistical theory. By using Lagrange equation, the conditional extremum problem of Equation (23) can be transformed into unconditional extremum problem:

(24) $$l(\beta ,\varepsilon ,\alpha )=\frac{1}{2}{\left\|\beta \right\|}^{2}+\frac{\lambda }{2}{\left\|\varepsilon \right\|}^{2}-\;\sum_{j=1}^{Z}\alpha_{j}(g(\omega_{i}•x_{j}+b_{i})-t_{j}-\varepsilon_{j})z$$

The simplified Equation (24) is:

(25) $$l(\beta ,\varepsilon ,\alpha )=\frac{1}{2}{\left\|\beta \right\|}^{2}+\frac{\lambda }{2}{\left\|\varepsilon \right\|}^{2}-\alpha (H_{2}\beta -T-\varepsilon )$$

$\alpha =[\alpha_{1},\alpha_{2},\ldots ,\alpha_{N}];\alpha_{1}\in R^{m}(j=1,2,\ldots N)$ stands for the Lagrange multiplier. Let the gradient of Equation (25) be 0:

(26) $$\{\begin{array}{c} \frac{\partial l}{\partial \beta }=\beta^{T}-\alpha H_{2}=0\\ \frac{\partial l}{\partial \varepsilon }=\lambda \varepsilon^{T}+\alpha =0\\ \frac{\partial l}{\partial \alpha }=H_{2}\beta -T-\varepsilon =0 \end{array}$$

If the input feature *N* is larger than any layer of neuron nodes, that is, *N* > *L* or *N* > *Z*, it can be obtained by Equation (26):

(27) $$\overset{}{\beta }={\left(\frac{I}{\lambda }+H_{2}^{T}H_{2}\right)}^{-1}H_{2}^{T}T$$

If the input feature *N* is less than any layer of neuron nodes, that is, *N* < *L* or *N* V< *Z*, it can be obtained by Equation (26):

(28) $$\beta =H_{2}{}^{T}{\left(\frac{I}{\lambda }+H_{2}H_{2}^{T}\right)}^{-1}T$$

If the input feature *n* is equal to the neuron node of each layer, that is, *N* = *L* = *Z*, then:

(29) $$\overset{}{\beta }=H_{2}^{+}T$$

where $H_{2}^{+}$ is the Moore Penrose generalized inverse of *H*_2_. The methods of calculating the Moore-Penrose generalized inverse of a matrix include: full rank decomposition method, inverse matrix method, singular value decomposition method (SVD). When $H_{2}^{T}H_{2}$ is nonsingular $H_{2}^{+}={\left(H_{2}^{T}H_{2}\right)}^{-1}H_{2}^{T}$, or when $H_{2}H_{2}^{T}$ is nonsingular $H_{2}^{+}=H_{2}^{T}{\left(H_{2}H_{2}^{T}\right)}^{-1}$.

Then the output of VTELM network is obtained as follows:

(30) $$y=H_{2}\beta$$

The basic steps of the final VTELM algorithm are:

(1) Firstly, we assume that the given training sample set is {*X*,*T*} = {*x_i_,t_i_*}(*i =* 1,2,…,*Q*). Hidden layer chooses the most appropriate activation function;

(2) Randomly select values for the weight *ω*_1_ and threshold *B*_1_ of the first hidden layer, and calculate the output matrix *H*_1_ of the first hidden layer by *H*_1_ = [*g*(*ω*_1_ × *x*)+*b*_1_];

(3) Randomly select values for the weight *ω*_2_ and threshold *B_2_* of the second hidden layer, and calculate the output matrix *H*_2_ of the second hidden layer by *H*_2_ = [*g*(*ω*_2_ × *H*_1_)+*b*_2_];

(4) Solve the connection weight *β* of the second layer and the output layer, and compare the input features (*N*) and number of neuron nodes size (*L*,*Z*);

(5) If *N* < *L* or *N* < *Z*, then $\beta =H_{2}{}^{T}{\left(\frac{I}{\lambda }+H_{2}H_{2}^{T}\right)}^{-1}T$;

(6) If *N* > *L* or *N* > *Z*, then $\beta ={\left(\frac{I}{\lambda }+H_{2}^{T}H_{2}\right)}^{-1}H_{2}^{T}T$;

(7) If these two values are equal: *β* = $H_{2}^{+}T$;

(8) Calculate the final output of the VTELM algorithm: $f(x)=g\left\{\omega_{2}•\left[g(\omega_{1}•x)+b_{1}\right]+b_{2}\right\}\beta$.

## 4. Experimental Results and Discussion

### 4.1. Processing of Copper Ore Spectral Data

The spectral data obtained by the copper ore sample through the spectral test has 973 dimensions. Due to the high dimensionality of copper ore and information redundancy, the network scale will be too complicated, the network training accuracy will decrease and the modeling effect will be worse. Therefore, principal component analysis (PCA) is used to simplify the spectral data, and the cumulative contribution rate can reach 99.8% when the principal component dimension is 15, and then the 15 dimensional principal component is used as the input of the network. 

**Figure 7:**
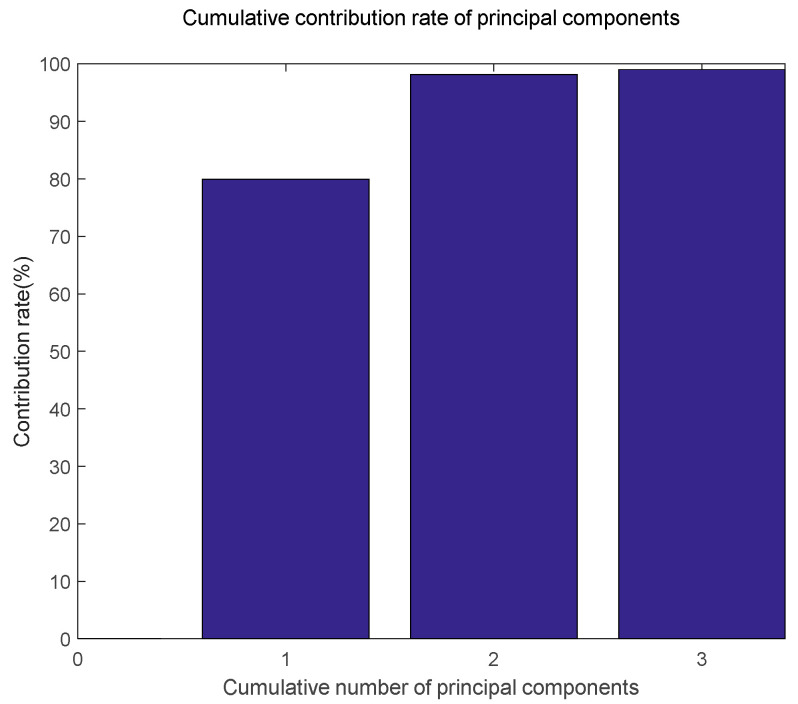
Cumulative contribution rate of principal component.

**Figure 8:**
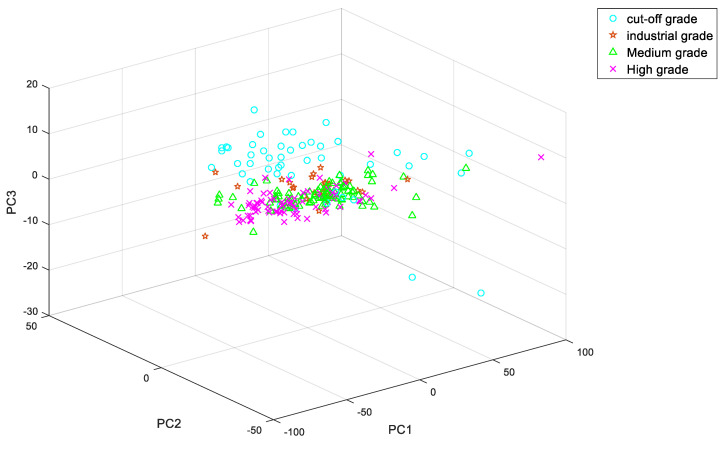
Spatial distribution map.

Figure 7 shows the cumulative contribution rate of the first three principal components and Figure 8 shows the spatial distribution of the first three principal components. Therefore, the initial data is compressed from 973 × 241 to 15 × 241, which reduces the interference of redundant information, improves the operation speed of the network and improves the accuracy of the model.

### 4.2. Neural Network Comparison

First, this paper used ELM, BP, and RBF to establish inversion models of the copper content in copper ore. Because of the small number of samples collected, in order to maximize the use of 251 sets of data to test different models, our three models have adopted cross validation method for 10 times of copper ore cross validation. Table 2 shows the test results of the three models, and compares the models in terms of time consumption (s), root mean square error (RMSE) and coefficient of determination (R^2^).

In Table 2, it can be seen that the root mean square error of ELM is 0.13653, which is the lowest of the three models. The root mean square error of BP is 0.15404, which is close to that of ELM, but the training time of BP is 12 times that of ELM. Therefore, the ELM model is most suitable for copper ore content detection.

### 4.3. Comparison of Copper Content Detection Models of Different Models

By comparing the experimental results of BP, ELM and RBF neural networks, we choose to use ELM to detect copper ore content. However, due to the limitation of ELM, VTELM model is proposed in this paper. In order to verify the superiority of VTELM, this paper uses ELM, TELM and VTELM to establish the inversion model of copper content in copper ore, and the experiment is simulated in MATLAB r2016a environment. Finally, by comparing the root mean square error, prediction time and coefficient of determination of the prediction set, the performance of the copper content detection model was evaluated.

**Figure 9:**
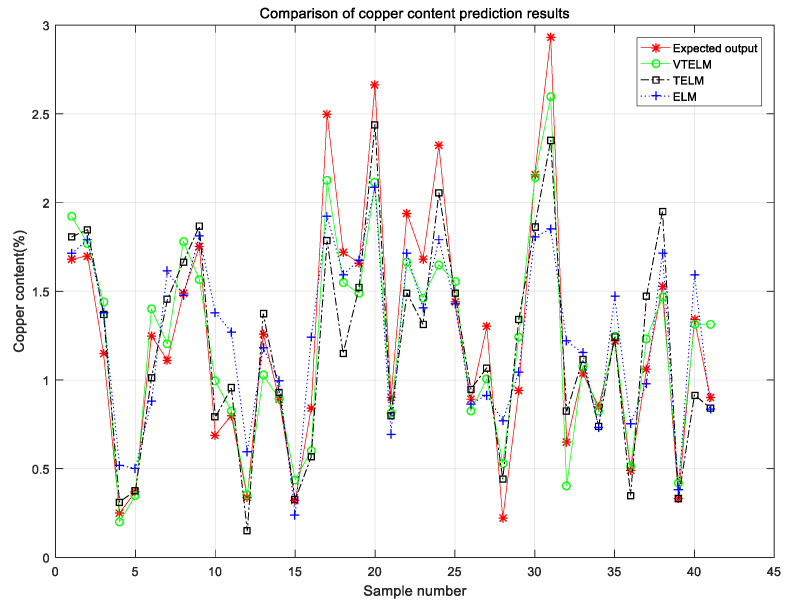
Experimental results of different models.

Figure 9 shows the single optimal cross validation results of Cu content models of ELM, TELM and VTELM for copper ores. Table 3 lists the performance of ELM, TELM and VTELM from the coefficient of determination (R^2^), root mean square error (RMSE), time consumption (S), and the number of hidden layer nodes. The results of specific analysis are as follows:

First of all, from the test results, compared with ELM and TELM, VTELM has the smallest root mean square error, which shows that the output value of VTELM is closer to the true value, so it can detect the Cu content in copper ore more accurately;

Secondly, compared with ELM and TELM, VTELM model has higher coefficient of determination, which indicates that VTELM has better goodness of fit and can be closer to the real value.

Third, the training time of TELM was significantly longer than that of ELM and VTELM, while the training time of ELM and VTELM was similar. This is because VTELM does not need to solve the connection weights of the two hidden layers and the threshold of the second hidden layer, and the time consumed is mainly used to solve the output matrix *β*.

Finally, for hidden layer nodes, ELM, TELM and VTELM all adopt a trial method, that is, hidden layer nodes iterate from 0 to 200 to find the best hidden layer nodes. For the copper ore Cu content detection model, ELM needs 11 hidden layer nodes, TELM needs 48 hidden layer nodes, and for VTELM, the first layer hidden layer nodes are 46, the second layer hidden layer nodes are 137. The more nodes in the hidden layer, the stronger the nonlinearity can be expressed by the neural network, which can describe the nonlinear characteristics of the fitting objective function more accurately, but the generalization performance will be reduced [40,41]. However, VTELM requires the most hidden layer nodes, and the generalization ability does not decrease. The results show that VTLEM has the highest accuracy and the strongest generalization ability.

In summary, VTELM has stronger generalization ability than TELM and ELM, the model is more accurate, and the calculation is more simple. Table 4 shows the detection method, chemical analysis method and instrument analysis method of VTELM based on copper ore spectral data, and compares them from three aspects of accuracy, time consumption and cost. It can be seen that the detection accuracy of the instrumental analysis method is low, and the chemical detection method has a high accuracy, but the cost is high and the time is long. The copper content detection method in copper ore proposed in this paper based on spectral data and VTELM algorithm has the advantages of short time-consuming, low cost, and high prediction accuracy, which can meet the needs of copper ore content identification.

In summary, the copper content detection model based on spectral data proposed in this paper is an improved two hidden layer extreme learning machine algorithms with variable hidden layer nodes, which has the optimal output matrix *β* and the weight matrix and hidden layer nodes of each hidden layer. Its generalization ability is better than ELM and TELM, its running speed is faster than TELM, and its coefficient of determination is the highest. Compared with the traditional instrument analysis method, this method is simple to operate, high in accuracy, fast in detection speed and low in cost.

## 5. Conclusions

In view of the shortcomings of traditional copper ore detection methods, this paper proposes a copper content detection model based on spectral data and VTELM, and validates the model with collected copper ore samples. First, we collected 241 copper ore samples, used chemical analysis to detect the copper ore to obtain the accurate copper content in the copper ore, and then performed a spectral test on it to obtain the initial spectral data. Since the initial spectral data has a large dimension and contains other useless information, PCA is used to reduce the dimension. Then, BP, ELM, and RBF were modeled separately using the reduced-dimensional spectral information. The modeling results showed that ELM has the fastest running speed and the smallest root mean square error. Due to the limitations of the ELM model, and in order to further improve the generalization ability of the model and reduce the difference between the predicted output of the model and the actual output, this paper proposes the VTELM model and conducts modeling experiments on ELM, TELM, and VTELM. By comparing the three models of ELM, TELM, and VTELM, VTELM has the highest coefficient of determination, the smallest error, and the running time is much lower than that of TELM, almost the same as ELM. Compared with the traditional methods, the copper content inversion model based on the reflectance spectrum and VTELM algorithm has the advantages of high accuracy, high reliability and fast detection speed. This paper provides a new idea for the detection method of the copper content in copper ore.

## Figures and Tables

**Table 1:** Parameters of SVC HR-1024.

Spectrometer Parameters	Parameter Value
Spectral Range	350–2500 nm
Internal Memory	500 Scans
Channels	1024
Spectral Resolution (FWHM)	≤3.5 nm, 350–1000 nm ≤9.5 nm, 1000–1850 nm ≤6.5 nm, 1850–2500 nm
Bandwidth (nominal)	≤1.5 nm, 350–1000 nm ≤3.6 nm, 1000–1850 nm ≤2.5 nm, 1850–2500 nm
Minimum Integration	1 millisecond

**Table 2:** Copper content detection models based on different neural networks.

Model Type	Time Consumption (s)	R^2^	RMSE
BP	0.202432	0.62688	0.15404
ELM	0.025085	0.62834	0.13653
RBF	0.062342	0.13653	1.6936

**Table 3:** Results of copper content detection model test.

Model Type	R^2^	RMSE	S	Number of Hidden Layer Nodes
ELM	0.74822	0.12112	0.020792	11
TELM	0.83589	0.075211	0.135268	48
VTELM	0.88309	0.055629	0.027361	46/137

**Table 4:** Comparison of detection methods.

Test Method	Detection Accuracy (%)	Time Consumed (h)	Cost Detection (yuan)
Instrument testing	73	3	About 400
Chemical method	99	70	About 21,000
VTELM	98.4	3	About 300
